# Effectiveness and cost effectiveness of interpersonal community psychiatric treatment (ICPT) for people with long-term severe non-psychotic mental disorders: a multi-Centre randomized controlled trial

**DOI:** 10.1186/s12888-021-03264-5

**Published:** 2021-05-19

**Authors:** Mark van Veen, Bauke Koekkoek, Steven Teerenstra, Eddy Adang, Cornelis L. Mulder

**Affiliations:** 1 Institute for Nursing studies, University of Applied Sciences, Heidelberglaan 7, 3584 CS Utrecht, the Netherlands; 2 Research Group for Social Psychiatry and Mental Health Nursing, University of Applied Science, Nijmegen and Pro Persona Mental Health Services, Arnhem, the Netherlands; 3 Department for Health Evidence, section Biostatistics, Radboud University Medical Center, Radboud Institute for Health Sciences, Nijmegen, the Netherlands; 4 Department of Psychiatry, Epidemiological and Social Psychiatric Research Institute, Erasmus MC, Rotterdam, the Netherlands; 5 Psychiatric Institute, Rotterdam, the Netherlands

**Keywords:** Effectiveness, treatment, Nursing

## Abstract

**Background:**

Long-term community mental health treatment for non-psychotic disorder patients with severe mental illness (SMI) who are perceived as difficult by clinicians, is poorly developed and lacks a structured, goal-centred approach. This study compares (cost-)effectiveness of Interpersonal Community Psychiatric Treatment (ICPT) with Care As Usual (CAU) on quality of life and clinician perceived difficulty in the care for non-psychotic disorder SMI-patients. A multi-centre cluster-randomized clinical tria was conducted in which Community Mental Health Nurses (Clinicians) in three large community mental health services in the Netherlands were randomly allocated to providing either ICPT or CAU to included patients. A total of 56 clinicians were randomized, who treated a total of 93 patients (59 in ICPT-group and 34 in CAU-group).

**Methods:**

Primary outcome measure is patient-perceived quality of life as measured by the Manchester Short Assessment of Quality of Life (MANSA). Secondary outcome measures include clinician-perceived difficulty, general mental health, treatment outcomes, illness management and recovery, therapeutic relationship, care needs and social network. Patients were assessed at baseline, during treatment (6 months), after treatment (12 months) and at 6 months follow-up (18 months). Linear mixed-effects models for repeated measurements were used to compare mean changes in primary and secondary outcomes between intervention and control group of patients over time on an intention to treat basis. Potential efficiency was investigated from a societal perspective. Economic evaluation was based on general principles of a cost-effectiveness analysis. Outcome measures for health economic evaluation, were costs, and Quality Adjusted Life Years (QALYs).

**Results:**

Half of the intended number of patients were recruited. There was no statistically significant treatment effect found in the MANSA (0.17, 95%-CI [− 0.058,0.431], *p* = 0.191). Treatment effects showed significant improvement in the Different Doctor-Patient Relationship Questionnaire-scores and a significant increase in the Illness Management and Recovery–scale Client-version scores). No effects of ICPT on societal and medical costs nor QALYs were found.

**Conclusions:**

This is the first RCT to investigate the (cost)-effectiveness of ICPT. Compared with CAU, ICPT did not improve quality of life, but significantly reduced clinician-perceived difficulty, and increased subjective illness management and recovery. No effects on costs or QALY’s were found.

**Trial registration:**

NTR 3988, registered 13 May 2013.

**Supplementary Information:**

The online version contains supplementary material available at 10.1186/s12888-021-03264-5.

## Background

Caring for patients with severe a mental illness (SMI) in long-term community mental health care has been, and still is, one of the major challenges for mental health systems. These SMI may have a low prevalence, the impact on patients, families and societies is huge [1].

Thornicroft et al. define community mental health care for SMI-patients as promoting mental health by being accessible, focusing on the patient’s goals and strengths, working evidence based and recovery-oriented and supporting network and services to get involved in the treatment [2]. Community mental health nurses (CMHNs) play an important role in community mental health care. Especially the care for patients with non-psychotic SMI (e.g. personality disorders) is an area in which the effectiveness of their interventions is unknown [3]. A good therapeutic relationship is very important for positive patient outcomes, yet methods to develop and maintain this relationship are poor, a recent systematic review showed [4].

There are, however, some interventions that are worth mentioning. First is the Boston Psychiatric Rehabilitation (PR) Approach which showed effectiveness in supporting patients to reach self-formulated rehabilitation goals and enhancing societal participation, yet without effects on quality of life, need for care and functioning [5]. Second is Illness Management and Recovery (IMR) that aims to improve illness self-management and achieving clinical and personal recovery. A recent trial showed no significant effect on clinical and personal recovery at the one-year follow-up [6]. Third is Structured Clinical Management (SCM), an evidenced based approach that enables generalist mental health clinicians to work effectively with patients with personality disorders. It provides a systematic approach and is based on case management and advocacy support [7].

In the Netherlands, long-term treatment of people with non-psychotic severe mental illness (SMI) is frequently offered in secondary mental health services, yet hampered by a lack of methodical underpinning and data on effectiveness [8]. Patients with depression, anxiety and substance abuse disorders, often combined with a personality disorder, may be treated for long periods (years on end), and service use may be substantial [9]. Treatment in the Netherlands is mostly provided by non-academic clinicians, mainly community mental health nurses (CMHNs) or social workers, who may perceive these patients as difficult [10]. Perceived patient difficulty is highly correlated with long-term and intensive care use [11, 12], as well as iatrogenic dependency [10].

This treatment, or Care As Usual (CAU) lacks an empirical and theoretical base and may increase dependency and repeated crises through its ad-hoc character [13] and absence of clear goals [14]. Without a clear frame, this CAU may turn into boundless long-term care, which may lead to high care use and dependency of the patient [15]. CAU is poorly described and investigated [9] and many clinicians experience a lack of a solid theoretical base from which to understand the mental disorder and its possible treatment. Interpersonal Community Psychiatric Treatment (ICPT) [16] was developed as an alternative for CAU.

The goal of this multi-centre randomized controlled trial was to compare the effectiveness and cost-effectiveness of ICPT to CAU in the treatment of people with aforementioned long-term severe non-psychotic mental disorders. Our primary hypothesis was that ICPT is more effective in improving patients’ quality of life. Secondary hypotheses were that ICPT is 1) more effective in decreasing clinicians’ perception of patients as ‘difficult’, and 2) that ICPT is more effective in discharging patients to a lower level of care (i.e. general mental health care instead of specialised mental health care) and 3) that ICPT is more cost-effective in reaching these goals than CAU. A promising pilot study [9] of 36 patients was done earlier. More detailed information can be found in our study protocol [17], that was published earlier.

## Method

### Design and patients

A multi-centre cluster randomized controlled trial in three large mental health services, which provided both inpatient and outpatient care, in which clinicians (mostly community mental health nurses) were randomly allocated to providing either ICPT or CAU, for a 12 months intervention period and a 6 months follow-up period.

The inclusion criteria for clinicians were:

- having an individual caseload of 5 or more patients with a non-psychotic disorder
- willing to be randomized to either the experimental ICPT-condition or CAU

Each clinician selected 5 patients to collect data from. The inclusion criteria for patients were:

- having a non-psychotic disorder
- being aged 18–65 years and being able to understand and communicate in Dutch
- receiving long-term treatment (2 years or more) or having high care use (1 or more outpatient contact per week or 2 or more crisis contacts per year or 1 or more inpatient admission per year

Exclusion criteria for patients were the presence of a psychotic, bipolar I or cognitive disorder and a lack of skill in understanding of, or communication in Dutch language.

Patients were informed about the study and were invited to participate. An invitation letter with attached information about the research was signed by the clinician, and sent by the department’s management. Patients who expressed their willingness to participate were either contacted by their clinician or the research team directly.

### Trial registration and ethical approval

This study was approved by a certified Medical Ethics Review Committee, The Clinical Research Centre Nijmegen (CRCN), in The Netherlands (Ref:44744.091.13) and the trial is registered (NTR:3988). Registered 13 May 2013, https://www.trialregister.nl/trial/3822

### Experimental and control conditions (treatments)

#### Interpersonal Community Psychiatric Treatment (ICPT)

The treatment under investigation was Interpersonal Community Psychiatric Treatment (ICPT), which aims to help patients to become more actively involved in their treatment process to reach a higher perceived quality of life. ICPT focuses on the interaction between patient, their social system and the patient’s responsibility for his or her own recovery. On the team level, ICPT supports clinicians by supervision to maintain treatment integrity.

ICPT is based on the interaction between patient, his social system and the clinician. It uses the perspective of learned ineffective illness behaviour [9] by both clinicians and patients. ICPT uses a general treatment frame including: (a) a clear session structure (mutual agenda setting and session evaluation using an established instrument), (b) a 3-stage model (in line with the patient’s level of cooperation and acceptance of help, comprising of three stages: (I) optimization of the working alliance, (II) clarification of, and agreement on goals and tasks, and (III): improvement of mental and social functioning), (c) a therapeutic method/style appropriate to the stage where the patient is in, (d) constant monitoring of the interpersonal contact between patient and clinician, and (e) support of clinicians through regular supervision. The ICPT-elements are shown in Table 1. Participating clinicians received a 4-day training program in the ICPT-group, over 4–6 weeks’ time. The intervention has been described in more detail before [17].

**Table 1:** ICPT-elements

1	Identifying treatment phase	Identification of stage 1 (alliance), 2 (goal setting), 3 (working)
2	Setting agenda	Joint agenda setting for the session
3	Looking back	Looking back at the previous session to maintain a course
4	Clarifying expectations	Matching mutual expectations of the session
5	Inventory of problems and needs	Inventory of needs according to structured instrument (CANSAS)
6	Setting goals	Goal setting based upon needs
7	Negotiating goals	Negotiating suitability and ranking order of goals
8	Working towards goals	Active working on goals, using structured methods
9	Using SRS-forms	Collection of structured session feedback
10	Using stage-specific methods	Using methods that fit the treatment phase

### Treatment integrity in ICPT

Treatment integrity in the ICPT-group was monitored by supervision every three to 4 weeks (with a total of 15 sessions) and by evaluating randomly selected audiotapes of treatment sessions. This was done by an independent rater (a Master-level student familiar with ICPT) masked to treatment condition, who assessed whether the tape was CAU or ICPT, and to which extent ICPT-elements were used (session structure, the 3-stage model and the therapeutic method or style). Additionally, clinicians scored their use of ICPT-elements in a session using a so-called ICPT-form – a checklist of the number of ICPT-elements used in each face-to face contact. The order of the checklist followed the chronological order of the treatment stages in ICPT. The scoring schedule rated the different elements in such a way that, regardless of the treatment stage, scores varied between 4 and 10, with a higher score indicating a higher degree of treatment integrity [9].

#### Care as usual (CAU)

The active control group was Care As Usual (CAU), which was a low-structured treatment/care consisting of biweekly outpatient contacts with a clinician, in which daily issues were discussed [8].

### Assessments

All instruments used in the study and their psychometric properties have been described in the study protocol, published earlier [17].

We used two quality of life outcomes. The MANSA was used for our clinical analysis, whereas the EQ-5D was used in the clinical and economic evaluation of health care, for our cost-effectiveness analysis.

#### Baseline

The first step in the baseline assessment was a structured diagnostic interview. Axis I disorders were assessed by use of the Mini Neuropsychiatric Interview (MINI Plus) [18]. The Structured Interview for DSM-IV (SIDP-IV) [19] was conducted only when the Standardized Assessment of Personality – Abbreviated Scale – Self Report (SAPAS-SR) [20] was positive.

#### Primary outcome

The primary outcome was quality of life, measured on patient level with the Manchester Short Assessment of Quality of Life (MANSA) [21]. It is the single most used quality of life instrument for patients with severe mental illnesses. Is it’s a 16-item patient-rated instrument with good psychometric properties.

#### Secondary outcomes

Secondary outcomes were a) the Difficult Doctor-Patient Relationship Questionnaire (DDPRQ), an 11-item instrument that assesses problems in the relationship between patient and clinician from the clinician perspective including the perceived difficulty (PD) as a single question about the clinician’s perceived difficulty in patient treatment [22], b) the Health of National Outcome Scale (HONOS) [23], that assesses overall mental functioning by the clinician perspective; (c) the Outcome Questionnaire (OQ-45) [24] that broadly assesses treatment outcomes from the patient perspective; (d) the Illness Management and Recovery scale (IMR) [25] that measures the extent of the patient’s management of serious mental illnesses from both patient (IMR-Patient) and clinician perspective (IMR-Clinician); (e) the Scale To Assess the Therapeutic Relationship (STAR) [26] that measures the quality of the therapeutic relationship between patients and clinicians, from both patient and clinician perspective; (f) the Camberwell Assessment of Need Short Appraisal Schedule (CANSAS) [27] that assesses care needs from both patient and clinician perspective; (g) the Social Network Map (SNM) [28] that assesses patient-perceived quantity and quality of the patient’s social network; (h) referral to lower intensive mental health services; (i) the EuroQol-5D (EQ-5D) [29] that measures health-related quality of life based on the patient perspecive, on the basis of which quality-adjusted life-years (QALYs) can be calculated and (j) the Trimbos/iMTA questionnaire for Costs associated with Psychiatric Illness (TiC-P) [30] that measures direct costs of medical treatments based on the patient perspective.

### Randomization

**Fig. 1:**
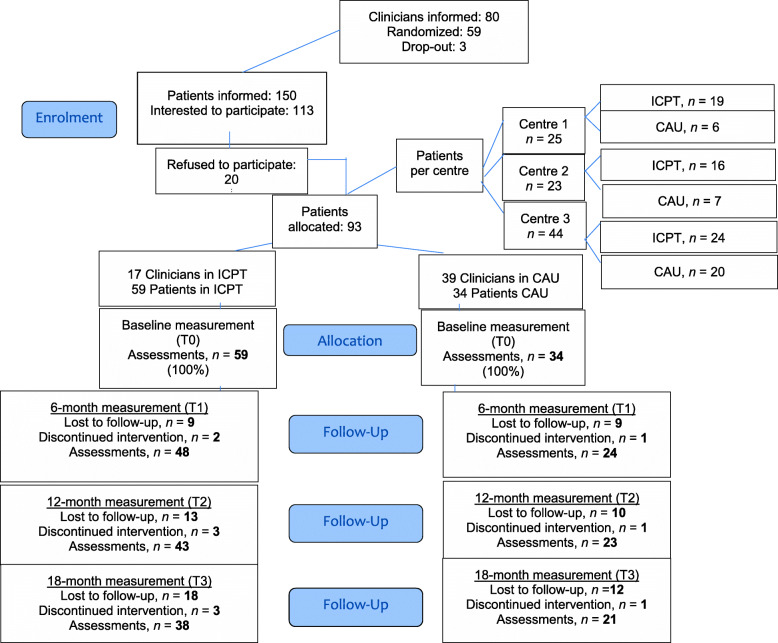
Consort diagram

Clinicians were randomized to either ICPT or CAU, stratified by unit within mental health service. The allocation sequences were generated with an automated algorithm, using a random sequence generation. This was done by a statistician at the Radboud University Medical Centre in the Netherlands who was not directly involved in the study. There was no possibility of blinding, since the ICPT-clinicians were trained, and patients knew they were in the ICPT-group. See Fig. 1 for the CONSORT-flow diagram.

### Sample size calculation

The sample size calculation was based on the primary outcome variable, quality of life as measured with the MANSA. With 36 clinicians and 5 patients per clinician (180 patients in total, 90 in the ICPT group and 90 in the CAU group), our study aimed for 80% power to detect an effect size of 0.3, assuming conservatively an intra-clinician correlation (ICC) of 0.10, a correlation between baseline and follow-up measurement of 0.5 for clinicians and 0.8 for patients.

### Procedures

After randomization, participating clinicians (both ICPT and CAU-condition) approached their own patients meeting the inclusion criteria and invited them to participate. When a patient agreed, he or she was contacted, and an appointment was made for a face-to-face contact with a researcher. In this contact, an explanation of the study was provided, written informed consent was obtained, and the baseline assessment was completed. Follow-up measurements were at 6 (intermediate), 12 (end of treatment period) and 18 months, all by telephone or face-to-face (when requested by the patient).

### Statistical analyses

Linear mixed-effects models for repeated measurements were used to compare mean changes in primary and secondary outcomes between the intervention and control group over time. The estimates in this multilevel analysis do not have to correspond to the observed results, because they are corrected for correlation of measurements over time and for correlation of patients within a clinician. Since after randomization a significant difference in level of education of patients was observed between experimental and control group, and the intervention implicitly depends on ability to self-reflect, education (low vs non-low) was corrected for in the analyses. Differences in ethnicity, marital status, and working status were not considered to be of such magnitude to confound the outcomes. Effect sizes were calculated as the estimated difference between groups at 12 months divided by the (pooled) standard deviation at baseline. The level for a statistically significant *p*-value was set at *p* < 0.05, but all *p*-values < 0.1 are reported. Intra-clinician and intra-patient ICC describing the correlation of patients within a clinician and the correlation of measurements within a patient, respectively, were estimated as $IC{C}_{professional}=\frac{\sigma_{professional}^2}{\sigma_{professional}^2+{\sigma}_{client}^2}$ and $IC{C}_{client}=\frac{\sigma_{professional}^2+{\sigma}_{client}^2}{\sigma_{professional}^2+{\sigma}_{client}^2+{\sigma}_{error}^2}$, where ${\sigma}_{professional}^2$ is the variance of the random intercept at clinician level, ${\sigma}_{client}^2$ is the variance of the random intercept at patient level, and ${\sigma}_{error}^2$ is the variance of the residuals [31]. Missing values were handled under the missing-at-random assumption in the linear mixed model analyses. We specifically did not perform a post-hoc analysis based on the observed effect and observed variances because it does not adress the problem of a possible type II-error [32].

Outcome measures for the economic evaluation, considering the 18-months period of evaluation, were costs, quality of life and quality adjusted life years (QALYs). On patient level, volumes of care were measured prospectively using TiC-P part I, administrative data. Cost items included were number of outpatient contacts, home visits, number and length of hospitalisations, but also ER/casualty department-visits. Productivity losses for patients (sick leave) were estimated using TiC-P part II. To measure the health-related generic quality of life of patients the EQ-5D was used.

For QALYs, regression models with cluster robust standard errors were used to the determine treatment effects. The cost variable was analysed by a generalised linear model with a log link function and gamma distribution. Here also cluster robust standard errors were applied. All models included the same set of covariates: sex, centre, education and age at baseline. The cost and QALY variables were presented with estimated marginal means. The level for a statistically significant *p*-value was set at *p* < 0.05. A Net Monetary Benefit (NMB) approach was used for the economic evaluation. The NMB depicts the difference in effects between the ICPT and the CAU-group multiplied with the Willingness to Pay (WTP) for a QALY minus the difference in costs between these treatment groups. When the NMB is larger than zero, the intervention is cost-effective. These NMB regression results can also be used to obtain a cost-effectiveness acceptability curve (CEAC) by plotting 1 minus p divided by 2 against a range of WTPs where p is the *p*-value from the coefficient on the treatment dummy variable (the divisor of two is employed because the acceptability curve is equivalent to a one-sided test).

## Results

Figure 1 (flowchart) shows that of 80 clinicians informed, 56 clinicians were recruited and randomized from August 2014 to August 2016. Two clinicians dropped out, one just before the start of the training and one immediately after the training. Recruited clinicians were predominantly women, working for at least 5 y within their mental health service. Clinicians were equally distributed across clusters. Then, 150 patients were informed, of whom 113 were initially interested to participate. Of those interested, 20 refused to participate: 8 because they were in the CAU-condition (but desired to participate in the ICPT-condition), 12 patients because they were in the ICPT-condition (but were unwilling to receive a new treatment or to assess questionnaires). In the end, only half of the planned number of patients were recruited. Of the 93 patients, 30 were lost to follow-up and 3 discontinued the intervention. Reasons for loss to follow-up were: physical illness or having quit treatment. Reasons for discontinued intervention were: lack of motivation for participation in the study or for further treatment. Two clinicians dropped-out due to change of work setting during treatment period. Table 2 shows baseline characteristics of the two groups, Table 3 shows the change from baseline to the end of the study, per 6 months.

**Table 2:** Socio-demographic and clinical characteristics at baseline of the ICPT-patients

Socio-demographic characteristics	ICPT (***N*** = 59)	CAU (***N*** = 34)	***p***
Age: mean (SD)	37(17.5)	41(12.7)	0.17
Sex: %, (*n)*			0.41
Female	72.9(43)	64.7(22)	
Male	27.1(16)	35.3(12)	
Ethnicity: %, (*n)*			0.22
Dutch	92.2(47)	73.5(25)	
Other	7.8(4)	26.5(9)	
Marital status: %, (*n)*			0.73
Married	20.3(12)	14.7(5)	
Unmarried	66.1(39)	58.8(20)	
Unknown	13.6(8)	26.5(9)	
Working status: %, (*n)*			0.58
Employed	16.9(10)	11.8(4)	
(temporarily) disabled	27.1(16)	38.2(12)	
Volunteer	11.9(7)	14.7(5)	
Looking for job	10.2(6)	5.9(2)	
Other	33.9(20)	29.4(10)	
Education: %, (*n)*			0.01
Primary education	3.4(2)	11.7(4)	
Secondary education	54.2(32)	44.1(15)	
Tertiary education	25.4(15)	35.3(12)	
Unknown/other	10(17.0)	8.8(3)	
Income: %, (*n)*			0.55
Salary	11.9(7)	11.8(4)	
Social benefit	47.5(28)	58.8(20)	
Student grant	8.5(5)	–	
Other	32.1(19)	29.4(10)	
**Clinical characteristics MINI Plus**			
Axis I: %, (*n)*			
Depressive disorder	20.7(12)	12.5(4)	0.33
Anxiety disorder	20.3(12)	8.8(3)	0.15
Alcohol abuse	10.2(6)	8.8(3)	0.84
Substance abuse	22(13)	20.6(7)	0.95
No or other diagnoses	26.8(16)	49.3(17)	–
**Clinical characteristics SIDP-IV**			
Axis II: %, (*n)*			
*Cluster A*			
Paranoid, schizoid, schizotypal PD	–	–	–
Cluster B			
Borderline PD	15.3(9)	14.7(5)	0.69
*Cluster C*			
Avoidant PD	11.9(7)	11.9(4)	0.69
Dependant PD	8.5(5)	8.8(3)	0.69
Obsessive-compulsive PD	6.8(4)	5.9(2)	0.93
No or other diagnoses	57.5(34)	58.7(20)	–

**Table 3:** Outcomes per measurement and estimated change from baseline

Type of outcome	Instrument		ICPT (***N*** = 59)				CAU (***N*** = 34)	
Quality of life	*Patient-rated*	Mean (SD)	*Change from baseline per 6 months estimate*, ([95%-CI])	*p-value*		Mean (SD)	*Change from baseline per 6 months estimate*, ([95%-CI])	*p-value*
	MANSA		0.082, ([0.023,0.142])	0.007	MANSA		0.025, ([−0.050,0.101])	0.511
	Baseline	4.1(0.8)			Baseline	4.8(0.8)		
	6 months	3.9(0.7)			6 months	4.6(1.1)		
	12 months	4.4(0.8)			12 months	4.6(0.8)		
	18 months	4.2(0.7)			18 months	4.8(0.8)		
Clinician-perceived patient difficulty	*Clinician-rated*	*Clinician-rated*						
	DDPRQ		−0.473, ([− 0.879,-0.050])	0.029	DDPRQ		−1.297, ([−1.881,-0.713])	< 0.001
	Baseline	23.8(4.8)			Baseline	22.9(4.7)		
	6 months	24.2(3.9)			6 months	20.6(4.9)		
	12 months	21.7 (4.5)			12 months	21.7(4.6)		
	18 months	22.3(3.7)			18 months	19.2(5.4)		
	PD		− 0.21, ([− 0.361,-0.074])	0.003	PD		−0.311, ([− 0.507,-0.114])	0.002
	Baseline	3.3(1.3)			Baseline	3.0(1.7)		
	6 months	3.2(1.6)			6 months	1.8(1.1)		
	12 months	2.4(1.3)			12 months	2.3(1.6)		
	18 months	2.7(1.3)			18 months	2.3(1.2)		
General Mental Health	*Patient-rated*	*Patient-rated*						
	HONOS		−1.056, ([− 1.480,-0.632])	< 0.001	HONOS		− 1.180, ([− 1.763,-0.597])	< 0.001
	Baseline	12.1(4.6)			Baseline	10.5(5.4)		
	6 months	9.7(3.9)			*6 months*	8.9(4.8)		
	12 months	8.1(4.6)			*12 months*	7.8(5.4)		
	18 months	9.1(4.9)			*18 months*	8.4(4.4)		
Treatment	*Patient-rated*	*Patient-rated*						
	OQ45		− 0.049, ([− 0.084,-0.015])	0.005	OQ45		−0.042, ([− 0.086,0.002])	0.065
	Baseline	81.7(23.7)			*Baseline*	65.1(27.5)		
	6 months	83.2(22.5)			*6 months*	65.0(31.3)		
	12 months	75.4(24.8)			*12 months*	64.1(22.2)		
	18 months	72.7(24.5)			*18 months*	59.7(26.8)		
Illness management and Recovery	*Patient-rated*	*Patient-rated*						
	IMR-Patient		0.037, ([0.002,0.073])	0.036	IMR-Patient		− 0.023, ([− 0.073,0.027])	0.369
	Baseline	3.2(0.4)			Baseline	3.3(0.4)		
	6 months	3.3(0.4)			6 months	3.3(0.4)		
	12 months	3.4(0,4)			12 months	3.4(0.4)		
	18 months	3.3(0.4)			18 months	3.3(0.3)		
	*Clinician-rated*	*Clinician-rated*						
	IMR Clinician		0.130, ([0.078,0.182])	< 0.001	IMR-Clinician		0.124, ([0.051,0.197])	0.001
	Baseline	3.1(0.3)			Baseline	3.3(0.4)		
	6 months	3.3(0.7)			6 months	3.2(0.6)		
	12 months	3.5(0.3)			12 months	3.5(0.4)		
	18 months	3.4(0.5)			18 months	3.5(0.4)		
Therapeutic relationship	*Patient-rated*	*Patient-rated*						
	STAR-Patient		0.121, ([− 0.458,0.701])	0.681	STAR-Patient		− 0.499, ([−1.223,0.225])	0.176
	Baseline	37.5(6.2)			Baseline	38.9(6.3)		
	6 months	37.2(5.9)			6 months	38.4(5.5)		
	12 months	38.7(5.6)			12 months	36.3(10.4)		
	18 months	38.2(5.1)			18 months	38.2(7.0)		
	*Clinician-rated*	*Clinician-rated*						
	STAR-Patient		0.094, ([−0.369,0.557])	0.689	STAR-Patient		0.137, ([− 0.049,0.684])	0.620
	Baseline	37.5(6.2)			Baseline	39.0(3.9)		
	6 months	37.2(5.9)			6 months	39.7(3.2)		
	12 months	38.7(5.6)			12 months	39.7(4.3)		
	18 months	38.2(5.1)			18 months	41.3(3.9)		
Care Needs	*Patient-rated*							
	CANSAS-Patient, unmet needs		−0.260, ([− 0.413,-0.108])	< 0.001	CANSAS-Patient, unmet needs		− 0.424, ([− 0.615,-0.233])	< 0.001
	Baseline	2.3(2.6)			Baseline	1.4(1.7)		
	6 months	1.8(2.2)			6 months	0.5(1.0)		
	12 months	1.4(1.9)			12 months	0.4(0.8)		
	18 months	1.4(2.0)			18 months	0.4(1.1)		
	Clinician-rated	*Clinician-rated*						
	CANSAS-Clinician unmet needs		−0.604, ([− 0.785,-0.422])	< 0.001	*CANSAS-Clinician unmet needs*		−0.768, ([− 0.981,-0.555])	< 0.001
	Baseline	2.5(2.8)			Baseline	2.4(2.3)		
	6 months	1.3 (2.0)			6 months	0.5(1.2)		
	12 months	0.7(1.4)			12 months	0.4(1.1)		
	18 months	0.6(1.4)			18 months	0.2(0.7)		
Social Network	*Patient-rated*	*Patient-rated*						
	SNM, quality of social network		0.024, ([− 0.007,0.045])	0.128	SNM, quality of social network		0.031, ([− 0.011,0.074])	0.145
	Baseline	0.6(0.5)			Baseline	0.6(0.4)		
	6 months	0.7(0.4)			6 months	0.8(0.4)		
	12 months	0.8(0.5)			12 months	0.6(0.5)		
	18 months	0.7(0.4)			18 months	0.7(0.4)		
	SNM, quantity of social network		− 0.085, ([− 0.156,-0.013])	0.020	SNM, quantity of social network		0.002, ([− 0.087,0.092])	0.960
	Baseline	−0.3(1.1)			Baseline	− 0.4(0.9)		
	6 months	− 0.6(0.9)			6 months	− 0.4(1.3)		
	12 months	− 0.7(0.8)			12 months	− 0.6(0.7)		
	18 months	− 0.4(0.8)			18 months	− 0.4(0.9)		

### Patient characteristics

#### Primary outcomes

The change from baseline in quality of life as measured by the MANSA (primary outcome) was 0.007 (95%-CI from 0.023 to 0.142, *p* = 0.082). In the CAU-group it was 0.511 (95%-CI from − 0.050 to 0.101, *p* = 0.025).

Table 4 shows treatment effects of ICPT as compared to CAU, effect sizes and ICC’s over the full 18-month treatment period. There was no statistically significant treatment effect (*p* = 0.191) on the primary outcome variable, meaning that ICPT was not more effective than CAU in improving quality of life.

**Table 4:** Estimated effects of ICPT as compared to CAU

Type of outcome	Instrument	Treatment effect* at 18 months [95%-CI] (***p***-value)	Cohen’s D at 18 months	ICC patient	ICC professional
Quality of Life	MANSA	0.17, [−0.058,0.431] (0.191)	0.21	0.08	0.02
Clinician-perceived patient difficulty	DDPRQ	2.47, [0.556,4.387] **(0.012)**	0.51	0.06	1.00
Clinician-perceived patient difficulty	PD	0.28, [−0.362,0.922] (0.390)	0.22	0.07	1.00
General Mental Health	HONOS	0.37, [−1.520,2.267] (0.696)	0.08	0.51	1.00
Treatment	OQ-45	−0.02, [− 0.172,0.125] (0.752)	0.00	0.20	0.00
Illness management and Recovery	IMR-Patient	0.18, [0.015,0.349] **(0.033)**	0.45	0.29	0.00
Illness management and Recovery	IMR-Clinician	0.02, [−0.223,0.260] (0.881)	0.06	0.05	0.65
Therapeutic relationship	STAR-Patient	1.86, [−0.513,3.427] (0.124)	0.24	0.23	0.00
Therapeutic relationship	STAR-Clinician	−0.13, [−1.865,1.606] (0.880)	0.02	0.05	0.15
Care needs	CANSAS-Patient	0.49, [−0.152,1.134] 0.133	0.19	0.25	0.37
Care needs	CANSAS-Clinician	0.49, [−0.128,1.114] 0.120	0.18	0.15	0.12
Social Network	SNM-quality	−0.02, [− 0.169,0.126] 0.773	0.04	0.27	0.17
Social Network	SNM-quantity	−0.26, [− 0.548,0.024] 0.072	0.24	0.09	0.00

*Treatment effect at 18 months is the difference between ICPT and CAU in change from baseline to 18 months. This is the group x time estimate in the linear mixed model multiplied by the appropriate number of 6-months periods (so 1x the group x time estimate for 6 months, 2 x for the 12 months, 3 x for the 18 months)

Table 5 shows the estimated health economic effects in both groups and estimated effects in the ICPT-group.

**Table 5:** Estimated health economic effects in the both groups and estimated effects in the ICPT-group

Type of outcome	Instrument	ICPT (***N*** = 59)	CAU (***N*** = 34)	Estimated effect over time	Treatment effect
	*Patient rated*	Mean (95%-CI)^#^	Mean (95%-CI)#	[95%-CI]	*p*
QALYs NL	EQ-5D	1.09(0.991,1.194)	1.07(0.942,1.199)	0.02 [−0.194,0.238]	0.84
Medical costs (€)	TiC-P	1927(1374,2479)	1513(834,2193)	1.27 [0.747,2.170]*	0.38
Total costs (€)	TiC-P	2129(1525,2734)	1978(1298,2890)	1.27 [0.748,2.153]*	0.38

^#^estimated marginal mean

*to be interpreted as a ratio of cost (coefficient of the treatment dummy). Here the intervention costs are, after considering cluster effect and covariates 27% higher than the control condition

#### Secondary outcomes

A significant treatment effect was found in the DDPRQ (2.47, 95%-CI [0.556,4.387], *p* = 0.012), meaning that clinicians perceived patients as less difficult in the ICPT condition as compared to CAU. A significant treatment effect also was found in the IMR-Patient Scale (0.18, 95%-CI [0.015,0.349], *p* = 0.033. Other hypotheses about ICPT included improvement of the social network of patients, more discharges to a lower level of care and better cost-effectiveness, yet all other outcomes did not show statistically significant effects of ICPT.

##### Cost effectiveness

**Fig. 2:**
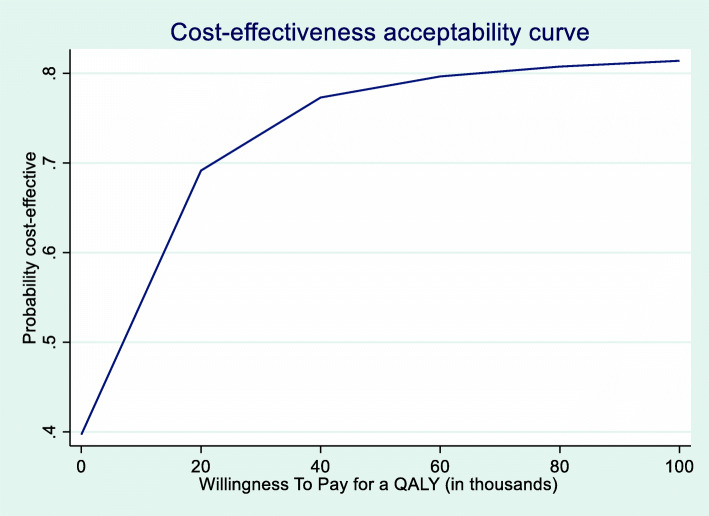
Cost-effectiveness acceptability curve

Neither costs (whether societal and medical costs), nor QALYs showed statistically significant treatment effects (Table 5). The ICPT-group had an estimated marginal mean cost of €2129 per patient. Looking at the point estimates, taking into account clustering and covariates, cost for ICPT were 27% higher (1.27, 95%-CI [0.75,2.17, *p* = 0.38]) than CAU. On the other hand, ICPT offered a higher mean incremental gain of 0.022 (EQ-5D; 95%-CI [− 0.194, 0.238]), *p* = 0.83) quality-adjusted life-years (QALYs) over 18 months. The cost-effectiveness acceptability curve (CEAC) (Fig. 2) showed that the cost-effectiveness improved if society is willing to pay more for a QALY. At about €70.000 the probability that ICPT was cost-effective, became 80%. This €70.000 is acceptable considering the threshold of €80.000 the Dutch Health Care Institute (Zorginstituut) uses to advise the minister on benefit package decisions. In fact, the more society is willing to pay for a QALY gained the higher the probability ICPT is a cost-effective approach compared to CAU.

##### Completers and dropouts

There were 38 ICPT-patients (out of 59) and 21 CAU-patients (out of 34) that completed the treatment. Significant treatment effects for completers, compared to all patients, were found in the DDPRQ and IMR-patient and in the CANSAS-patient version and CANSAS-clinician and the STAR-patient version (See Additional file 1 for details). Besides patients who were lost to follow-up or discontinued the intervention, a number of ICPT-clinicians was lost due to the cluster design of the RCT. Two clinicians quit their jobs early in the study and one clinician stopped just before the intervention started (but had received the 4-day training program), which resulted in a loss of 15 patients and an actual loss of 4 patients.

##### Treatment integrity

Only six audiotapes could be analysed and transcribed, due to a low number of recordings made by clinicians. From the analysis of the audiotapes we found that clinicians partially worked according to the ICPT-treatment model, yet we were unable to validly assess treatment integrity in this way.

ICPT-forms [9] were assessed by the ICPT-clinicians after each ICPT-sessions. We could analyse ICPT-forms of 10 participating ICPT-clinicians, for a total of 22 patients. The total number of completed ICPT-forms was 162 and average number of completed ICPT-forms per clinician was 7.34 (SD = 6.01). ICPT-scoring forms were assessed in 74% of the sessions. The number of ICPT elements used during ICPT showed a range of scores between 1 and 11, with a mean of 5.8 (SD 2.3). Mutual agenda setting was done in 66% of the sessions, whereas session evaluation was done in 55.6%. On all forms the identification of stage was scored. Most of the sessions were in the *improvement of mental and social functioning* stage (stage III, 90.7%). The use of a specific therapeutic method was scored in 56.2% of the sessions, with motivational interviewing (21.6%) and behavioural analyses (12.3%) as the most used therapeutic methods.

## Discussion

We conducted a cluster RCT on Interpersonal Community Psychiatric Treatment (ICPT) versus Care As Usual (CAU). This study did not find statistically significant treatment effect in the primary outcome variable, quality of life as assessed by the MANSA. Significant beneficial treatment effects were found in clinician-perceived patient difficulty, and patient-perceived illness management and recovery. No effects of ICPT on societal and medical costs or QALYs were found.

### Primary outcome measure

Quality of life has become an important outcome in health care as an indicator of treatment effectiveness and recovery [22, 27]. We aimed for an effect size of 0.3.Although the confidence interval (from − 0.058 to 0.431) did not exclude an effect size of 0.3, the point estimate suggests that the. Effect size is less than 0.3 but it is indecisive whether the effect is > 0. In line with this, we could not show that ICPT was statistically significantly better than CAU, although MANSA-scores increased numerically more in the ICPT-group than in the CAU-group.

### Secondary outcome measures

As in the pilot study [9], the professional-perceived therapeutic relationship increased in the ICPT condition compared to CAU. The clear structure, goal setting and working alliance may have contributed to that. Patient-rated illness management recovery increased in the ICPT-group. This is encouraging, even though ICPT had a different focus: not so much on managing one’s illness, but on increasing one’s positive interactions and daily activities [25].

The potential societal gains of ICPT were not substantial. ICPT provided no statistically significant efficiency gain since differences in both cost and QALY turned out to be insignificant. Whereas ICPT appeared somewhat (although not significantly) more expensive than CAU, due to higher medical costs, it was also somewhat more effective (yet neither significantly). Ultimately it was about a trade-off as can be inferred from the cost-effectiveness acceptability curve (CEAC). The Dutch Healthcare Institute uses a threshold of €80.000 per QALY gained. Taking uncertainty surrounding cost-effectiveness into account for ICPT it is slightly above 80% probable that it will be cost-effective.

### Treatment integrity

One of the ways in which we measured treatment integrity was by using standardized forms. Compared to the pilot study, in which these forms were also used [9], the mean score of ICPT-elements used here was overall lower than in the pilot study meaning that ICPT was more adequately applied in the pilot study [9]. A lack of a sufficient audio tapes made it impossible to rate recorded sessions, and assess treatment integrity through this method. To monitor and enhance treatment integrity, constant supervision during ICPT-treatment is important and should be applied systematically and on a regular basis to enhance treatment integrity [33]. Attendance of supervision sessions was sometimes low and keeping CHMNs motivate to remain focussed on the interpersonal element of ICPT was challenging. We may, therefore, hypothesize that the overall implementation was not optimal, given the limited supply of required ICPT-forms and audio-tapes by clinicians.

Treatment integrity, dropouts (on patient and clinician level) and inclusion numbers of this multi-centre cluster randomized RCT, showed how challenging it was to conduct this study. Finding three mental health services that wanted to participate was not easy at a time when mental health institutions were under public and political pressure to perform and CMNH’s had many patients in their caseloads and on waiting lists. As a result, we have strong indications that ICPT was not fully embraced within the organizations, teams and individual clinicians, despite their willingness to participate.

### Comparisons with other studies

Overall, there seems to be a lack of comparable studies, regarding effective interventions for non-psychotic SMI-patients in community mental health nursing. A recent study suggests that therapeutic alliance in mental health nursing is very important, but found that the evidence based methods to achieve that alliance are poor [4]. In the Netherlands, Structural Clinical Management (SCM) is used in outpatient treatment for personality disorders. SCM has been found be equally effective as other treatments such as Dialectical Behavioural Therapy (DBT) and Mentalisation Based Treatment (MBT) [7]. It may be used by general mental health clinicians, and like ICPT it works with a structured framework, yet has only been tested in a small population (i.e. borderline personality disorder), whereas ICPT serves a broader population. The aforementioned Boston Psychiatric Rehabilitation (PR) seems promising in terms of rehabilitation and participation and both PR and ICPT share the mutual agreed upon goal setting. PR though, has no specific focus on patient-perceived difficulty. Illness and Management Recovery (IMR) focusses on illness management whereas ICPT places more emphasis on the interpersonal relationship.

#### Strengths and limitations

The present study has some limitations. The required number of patients as defined in the sample size analysis (180 patients) was not reached, despite substantial efforts and instructions to support clinicians in recruiting suitable patients. There were difficulties in the implementation phase, e.g. recruiting clinicians and keeping them motivated to participate in this study.

Especially in the control group it turned out to be challenging to have patients recruited by clinicians, resulting in a low number of patients compared to the ICPT-group. We do not know whether selection bias occurred in the ICPT-group or CAU-group, respectively, but we know that patients did not want to participate due to lack of desire in a new treatment of unwillingness to fill out questionnaires over time. There is a loss of statistical power through missing follow-up data resulting from the 18-month follow-up. It must be noted that the completers-analysis is vulnerable to selection bias. Another limitation is the fact that we did not assess self-reported costs (e.g. transportation costs), regarding the cost effectiveness. The limitations of the present study are, however, balanced by a number of strengths. We performed a cluster randomized controlled trial, aiming to reduce the potential for contamination between treatment groups and we did include the number of clusters aimed for (36) and even exceeded that by 20 (56). Since the number of clusters is the driving factor for power, the loss of power due to not reaching the number of patients was substantially reduced. Finally, for the understanding of the effects of ICPT in a pragmatic, real-world setting and generalisability of the findings, the research was carried out in real-life practice with a heterogeneous group of patients.

## Conclusions

No significant treatment effect was found in the primary outcome: quality of life. Treatment effects were found on clinician perceived patient difficulty and on patient-perceived illness management and recovery, however, these were not corrected for multiple testing and should therefore be regarded as promising, not confirmative. Compared to CAU, ICPT was not cost-effective from a societal or medical perspective. Given the effects on clinician perceived patient difficulty, we recommend further developing and investigating ICPT as one of the interventions to work more successfully with patients with long term non-psychotic mental disorders.

## Supplementary Information

**Additional file 1: **Estimated effects of ICPT as compared to CAU for completers (ICPT *N* = 38, CAU *N* = 21)

## Data Availability

The datasets generated during and analysed during the current study are not publicly available, and we do not wish to share our data. But they are available from the corresponding author on reasonable request.

## Abbreviations

CANSAS: Camberwell Assessment of Need Short Appraisal Schedule
CAU: Care As Usual
CEAC: cost-effectiveness acceptability curve
CLINICIAN: Community Mental Health Nurse
DDPRQ: Difficult Doctor Patient Relation Questionnaire
EQ-5D: EuroQol-5D
HONOS: Health Nation Outcome Scale
ICPT: Interpersonal Community Psychiatric Treatment
IMR: Illness Management and Recovery
MANSA: Manchester Short Assessment of Quality of Life
MHS: Mental Health Services
MINI Plus: (Mini Neuro-psychiatric Interview)
OQ45: Outcome Questionnaire45
SAPAS: Standardised Assessment of Personality –Abbreviated Scale, Self-Report -SIDP-IV, Structured Interview for DSM-IV
SMI: Severe Mental Illness
SNM: Social Network Map
STAR: Scale to Assess the Therapeutic Relationship
Tic-P: Trimbos/iMTA questionnaire for Costs associated with Psychiatric Illness
