# Supplementary material for: Effectiveness and cost effectiveness of interpersonal community psychiatric treatment (ICPT) for people with long-term severe non-psychotic mental disorders: a multi-Centre randomized controlled trial
Source: BMC Psychiatry. 2021 May 19;21:261. doi: 10.1186/s12888-021-03264-5 (PMC8136177; doi:10.1186/s12888-021-03264-5)
Supplement: Supplementary file 1 — Additional file 1: Estimated effects of ICPT as compared to CAU for completers (ICPT N = 38, CAU N = 21) [file 12888_2021_3264_MOESM1_ESM.docx]

**Appendix I Estimated effects of ICPT as compared to CAU for completers (ICPT N=38, CAU N=21)**

| **Type of outcome** | **Instrument** | **Treatment effect* at 18 months [95%-CI]**  **(*p*-value)** |
| --- | --- | --- |
| Quality of Life | MANSA | 0.21, [-0,051,0.470]  (0.114) |
| Clinician-perceived patient difficulty | DDPRQ | 2.26, [0.113,4.402]  **(0.039)** |
| Clinician-perceived patient difficulty | PD | 0.35, [-0.280,0.981]  (0.267) |
| General Mental Health | HONOS | 1.12, [-0.948,3.197]  (0.282) |
| Treatment | OQ-45 | -0.01, [-0.174,0.164]  (0.952) |
| Illness management and Recovery | IMR-Patient | 0.91, [0.014,0.378]  **(0.035)** |
| Illness management and Recovery | IMR-Clinician | -0.01, [-0.281,0.269]  (0.964) |
| Therapeutic relationship | STAR-Patient | 0.85, [-1.608,3.380]  0.510 |
| Therapeutic relationship | STAR-Clinician | -3.48, [-5.475,-1.500]  **0.001** |
| Care needs | CANSAS-Patient | 0.89, [0.083,1.700]  **(0.031)** |
| Care needs | CANSAS-Clinician | 0.90, [0.135,1.673]  **(0.021)** |
| Social Network | SNM-quality | 0.06, [-0.097,0.217]  (0.543) |
| Social Network | SNM-quantity | -0.22, [-0.537,0.098]  (0.175) |

*Treatment effect at 18 months is the difference between ICPT and CAU in change from baseline to 18 months. This is the group x time estimate in the linear mixed model multiplied by the appropriate number of 6-months periods (so 1x the group x time estimate for 6 months, 2 x for the 12 months, 3 x for the 18 months).
